# Immunological characteristics of a recombinant alphaherpesvirus with an envelope-embedded *Cap* protein of circovirus

**DOI:** 10.3389/fimmu.2024.1438371

**Published:** 2024-07-16

**Authors:** Chenhe Lu, Haimin Li, Wenjing Chen, Hui Li, Jiayu Ma, Peng Peng, Yan Yan, Weiren Dong, Yulan Jin, Shiyue Pan, Shaobin Shang, Jinyan Gu, Jiyong Zhou

**Affiliations:** ^1^ MOA Key Laboratory of Animal Virology, Zhejiang University Center for Veterinary Sciences, Hangzhou, China; ^2^ College of Veterinary Medicine, Yangzhou University, Yangzhou, China; ^3^ State Key Laboratory for Diagnosis and Treatment of Infectious Diseases, First Affiliated Hospital, Zhejiang University, Hangzhou, China

**Keywords:** chimeric pseudorabies virus, circovirus, immunity, memory responses, IFN-γ

## Abstract

**Introduction:**

Variant pseudorabies virus (PRV) is a newly emerged zoonotic pathogen that can cause human blindness. PRV can take advantage of its large genome and multiple non-essential genes to construct recombinant attenuated vaccines carrying foreign genes. However, a major problem is that the foreign genes in recombinant PRV are only integrated into the genome for independent expression, rather than assembled on the surface of virion.

**Methods:**

We reported a recombinant PRV with deleted gE/TK genes and an inserted porcine circovirus virus 2 (PCV2) *Cap* gene into the extracellular domain of the PRV gE gene using the Cre-loxP recombinant system combined with the CRISPR-Cas9 gene editing system. This recombinant PRV (PRV-Cap), with the envelope-embedded Cap protein, exhibits a similar replication ability to its parental virus.

**Results:**

An immunogenicity assay revealed that PRV-Cap immunized mice have 100% resistance to lethal PRV and PCV2 attacks. Neutralization antibody and ELISPOT detections indicated that PRV-Cap can enhance neutralizing antibodies to PRV and produce IFN-γ secreting T cells specific for both PRV and PCV2. Immunological mechanistic investigation revealed that initial immunization with PRV-Cap stimulates significantly early activation and expansion of CD69^+^ T cells, promoting the activation of CD4 Tfh cell dependent germinal B cells and producing effectively specific effector memory T and B cells. Booster immunization with PRV-Cap recalled the activation of PRV-specific IFN-γ^+^IL-2^+^CD4^+^ T cells and IFN-γ^+^TNF-α^+^CD8^+^ T cells, as well as PCV2-specific IFN-γ^+^TNF-α^+^CD8^+^ T cells.

**Conclusion:**

Collectively, our data suggested an immunological mechanism in that the recombinant PRV with envelope-assembled PCV2 Cap protein can serve as an excellent vaccine candidate for combined immunity against PRV and PCV2, and provided a cost-effective method for the production of PRV- PCV2 vaccine.

## Introduction

Pseudorabies virus (PRV), as a representative member of *Alphaherpesviridae*, has a broad host spectrum, including domestic pigs, wild boars, cattle, sheep, goats, dogs, cats, mice, and rabbits (1–3). PRV infection results in great economic loss to the global pig industry (4, 5). Recently, a newly emerging variant of PRV (Type II) has been reported to be transmissible to humans, causing ocular, respiratory, or central nervous system diseases (6–8). Thus, PRV infection is a public health concern. Given that PRV deleting non-essential genes, i.e., TK, gE, gI, and gG genes, attenuate its virulence (9–14), thus, the classical PRV has been described to express foreign antigen proteins to explore the feasibility of PRV as a potential vaccine vector, such as GP5 protein of porcine reproductive and respiratory syndrome virus (PRRSV) (15), E2 protein of classical swine fever virus (CSFV) (16), foot-and-mouth disease virus VP1 protein (17), porcine parvovirus (PPV) VP2 protein (18), and Senecavirus A (SVA) VP2 protein (19). However, the immunoprotective mechanism of a combined vaccine against the newly emerging pseudorabies virus type 2 and porcine circovirus type 2 has not been reported.

Adaptive immunity, as an important immune mechanism, is composed of humoral immunity mediated by antigen-specific antibodies and cellular immunity, mainly composed of T cells (20). Effective humoral immune responses activated by exogenous pathogens or vaccine depend on the production of high-affinity antibodies (21). Germinal centers (GCs) are the microanatomical structures of clonal expansion and affinity maturation of B lymphocytes in secondary lymphoid organs after exposure to antigens (22). GCs perform positive selection on GC B lymphocytes to produce high-affinity antibodies by obtaining co-stimulatory signals from recruited T follicular helper cells (Tfh) (23, 24). Bcl6 is an important master regulator that drives the maturation of Tfh and GC B. Mature Tfh cells expressing CD40L bind to CD40 on the surface of GC B, and subsequently activate the NF-κB pathway to trigger the expansion of B cells (25–28). Tfh also secretes a variety of cytokines, mainly IL-21 (29), to promote the maintenance of GC B and the production of plasma cells. These signals together determine the fate of GC B and participate in humoral immune regulation (30).

In addition to humoral immunity, cell-mediated immunity also plays an important role in the defense against virus infections, including influenza virus (31, 32), Middle East respiratory syndrome coronavirus (33), severe acute respiratory syndrome coronavirus 2 (34–36), Hepatitis B virus (37), and human immunodeficiency virus (38). Cellular immunity not only reflects the antiviral effect during infection, but also can be used to evaluate the immune effect of vaccines (39). CD4 and CD8 T cells mediate adaptive cellular immune responses after maturation in the thymus and have important auxiliary effects in antigen-induced humoral immunity (40). Viruses can stimulate CD4 T cells to differentiate into a variety of subsets including Th1, Th2, Th17, and Tfh. Th1 CD4 T cells secrete cytokines, mainly including IFN-γ, TNF-α, and IL-2 (41–44). IFN-γ and TNF-α synergistically inhibit viral replication, while IL-2 promotes the expansion and activation of various T cells. Studies have shown that a single cytokine indicator is insufficient to accurately reflect immune protection and multifunctional T cells that simultaneously produce IFN-γ, IL-2, and TNF-α can provide a better evaluation of specific cellular immunity (45–48). At the same time, Th1 CD4 T cells activate cytotoxic T lymphocytes (CTLs) to release granzyme and perforin to clear infected cells by inducing programmed cell death (49, 50). Therefore, the comprehensive evaluation of cellular and humoral immunity is particularly important to understand antiviral immunity.

In this study, the Capsid protein (Cap) of PCV2 was selected to construct a recombinant virus using PRV type II as the vector. An efficient recombinant PRV with the Cap of PCV2 (PRV-Cap) was successfully rescued using a combination of the Cre-loxP recombinant system and the CRISPR-Cas9 gene editing system. The recombinant Cap-gE fusion protein was stably assembled in the viral envelope of PRV. PRV-Cap immunized mice showed 100% survival to lethal PRV and PCV2 attacks. The initial immunization of PRV-Cap activated the expansion of PRV and PCV2-specific T cells, promoting the activation of germinal B cells through CD4 Tfh cells to produce specific antibodies. The booster immunization of PRV-Cap recalled the activation of PRV-specific IFN-γ^+^IL-2^+^CD4^+^ T cells, IFN-γ^+^TNF-α^+^CD8^+^ T cells, and PCV2-specific IFN-γ^+^TNF-α^+^CD8^+^ T cells. Our data suggested that PRV-Cap can effectively produce specific effector memory T cells (Tem) and memory B cells (MBCs) and exhibits weak secondary memory response after virulent PRV and PCV2 infection.

## Materials and methods

### Cells and viruses

Human embryonic kidney cells (HEK293T, ATCC CRL-11268), African green monkey kidney cells (Vero, CCL-81), and PCV-free PK-15 cells (ATCC-CCL-33) were cultured in Dulbecco’s modified Eagle’s medium (DMEM; Gibco, America) and supplemented with 10% heat-inactivated fetal bovine serum (FBS; Gibco, America). PCV2 strains HZ0201, PCV2 strains ZJ/c, PRV type II (PRV II) strains Dx (virulent, PRV Dx virus), and HD/c (gE/TK deletion, PRV HD/c virus) were stored in our laboratory (51, 52).

### Antibodies and reagents

Mouse monoclonal antibodies (mAbs) against PCV2 Rep, PCV2 Cap, PRV gC, and PRV gD and rabbit pAbs to PRV VP5 were maintained in our laboratory (53, 54). Anti-β-actin (M1210-2) and rabbit anti-GFP (ET1602-7) polyclonal antibodies (pAbs) were obtained from Hangzhou HuaAn Biotechnology. Goat anti-mouse IgG H&L (10 nm Gold) was purchased from Abcam. Protein A/G PLUS-Agarose (sc-2003) was purchased from Santa Cruz Biotechnology. A UNIQ-10 Column Virus Genomic DNA Isolation Kit (CB94701427), Streptomycin sulfate (A610494-0050), and penicillin G sodium (A600135-0025) were purchased from Sangon Biotechnology. Phenol reagent for DNA Extraction (T0250) was purchased from Solarbio. ELISA kits for PRV gB antibody and PCV2 Cap antibody were purchased from Beijing Jinnuo Biotech Co., Ltd and Ringpu (Baoding) Biological Pharmaceutical Co. Ltd, respectively.

### Construction for Cap protein of PCV2 transfer and US2 CRISPR-Cas9 gene editing vectors

**Figure 1:**
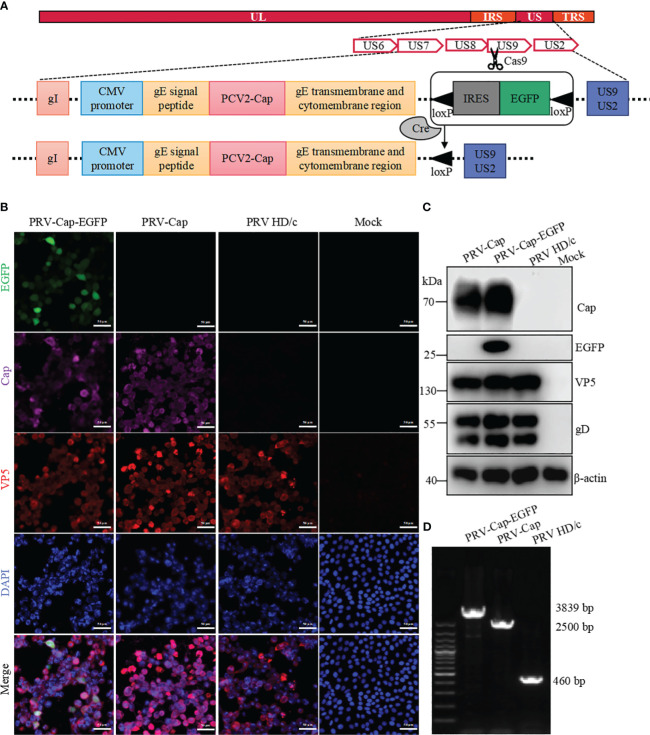
Generation of recombinant PRV with the Cap protein of PCV2. **(A)** The construction strategy of PRV-Cap. The PCV2 *Cap* gene was inserted into the *gE* extracellular region of parent PRV HD/c virus strain by homologous recombinant transfer vector, and then the fluorescent labeled *EGFP* gene was removed *in vitro* by the Cre-LoxP recombinant enzyme system to obtain the recombinant PRV with only the exogenous *Cap* gene. **(B)** IFA and **(C)** Western blotting assays of Cap-gE fusion protein expression in Vero cells inoculated with 1 MOI of PRV-Cap-EGFP, PRV-Cap, and PRV HD/c virus at 24 h post infection **(D)** Identification of inserting the *Cap* gene in Cap-EGFP and PRV-Cap by nucleic acid electrophoresis using gE-US7-9 primers.

The PRV envelope glycoprotein gE (US8) was selected as the insertion site for the PCV2 *Cap* fragment. Briefly, the signal peptide and transmembrane region of gE were predicted by the online software SignalP-5.0 Server and TMHMM Server v.2.0. The signal peptide sequence of gE contained the residues 1-430 in the extracellular region, the residues 431-453 in the transmembrane region, and the residues 454-579 in the intracellular region. The gE extracellular region residues 44-397 were replaced with PCV2 Cap to express a Cap-gE fusion protein. To enhance the expression of the Cap-gE fusion protein, the CMV promoter was inserted upstream of the gE signal peptide. Downstream of the fusion protein, an IRES sequence and the EGFP gene were added. On either side of the IRES-EGFP sequence, there were two loxP sites in the same direction for subsequent removal of the EGFP selection tag by the Cre enzyme. **Figure 1A** shows the skeleton of the Cap-gE homologous recombinant transfer vector that contains the sequence of CMV promoter, IRES, EGFP, and loxP sites. Primer pairs are listed in **Table 1** for the construction of the Cap-gE transfer vector. The sequence scan for CRISPR online website was used to design sgRNA for the PRV *US9* gene sequence, and two primer pairs of sgRNA sequences with scores greater than 1.0 were synthesized as follows: PX459-US9-sgRNA1-sense and antisense primers: 5’-CACCGCGGCTCGCTGGCCCTGCTGC-3’ and 5’-AAACGC AGCAGGGCCAGCGAGCCG-3’; PX459-US9-sgRNA2 sense and antisense primers: 5’-CACCGCCGTCCACCTGTGGATCCTG-3’ and 5’-AAACCAGGATCCACAGGTGGACGG-3’. The synthesized primers were used to connect the px459 plasmid cut by the *BbsI* enzyme.

**Table 1:** Primers used for the construction of Cap-gE transfer vector.

Groups		Sequence(5’→3’)
Homologous left arm	LgE-F plus	GAATTCGAGCTCGGTACCCACGTCGCCGGCAGCGCCGTCCTC
	LgE-R-CMV plus	TTGATTACTATTAATAACTAGGTCTCAACCCCGGTGTGTG
CMV promoter	CMV-F-LgE plus	CACACACCGGGGTTGAGACCTAGTTATTAATAGTAATCAATTAC
	CMV-R-gESP plus	CGCAGCAGAAAGGGCCGCATGGTGGCGATCTGACGGTTCACTAAACCAG
gE signal peptide	gESP-F-CMV plus	GTGAACCGTCAGATCGCCACCATGCGGCCCTTTCTGCTGC
	gESP-R-Cap plus	CGGGTGTTGAAGATGCCATTGGCCGAGGGACTCGGGACCTCGGTGAC
PCV2 HZ0201 Cap	Cap-F-LgE plus	AGGTCCCGAGTCCCTCGGCCAATGGCATCTTCAACACCCGCCTCTC
	Cap-R-RgE plus	ATCGCGTCGTCGCCGCCGCCAGGGTTAAGTGGGGGGTCTTTAAG
Insertion domain of gE	gEKB-F-Cap plus	AAGACCCCCCACTTAACCCTGGCGGCGGCGACGACGCGATCTAC
	gEKB-R-loxP plus	ATAACTTCGTATAGCATACATTATACGAAGTTATTTAAGCGGGGCGGGACA
IRES sequence	IRES-F-loxP plus	TGTATGCTATACGAAGTTATGCCCCTCTCCCTCCCCCCCCCCTAA
	IRES-R-EGFP plus	CTTGCTCACCATTGTGGCCATATTATCATCGTGTTTT
EGFP sequence	EGFP-F-IRES plus	AATATGGCCACAATGGTGAGCAAGGGCGAGGAGCTGT
	EGFP-R-loxP plus	ATAACTTCGTATAGCATACATTATACGAAGTTATCTACTTGTACAGCTC
Homologous right arm	RgE-F-loxP plus	TGTATGCTATACGAAGTTATATACCGGGAGAACCGGTG
	RgE-R plus	GACGGCCAGTGCCAAGCTTGTTGTGGACCCGCGCGAACATGGCG

### Rescue of recombinant virus

HEK293T cells were seeded on cell plates and were co-transfected with PRV HD/c virus genomic DNA, Cap transfer, and US9 CRISPR-Cas9 gene editing vectors by Jet prime transfection reagent (Polyplus-transfection, France) according to the manufacturer’s instructions. After being cultured at 37°C for 6 h, the resultant cells were replaced with DMEM medium containing 2% FBS for another 48 h. The supernatant of the co-transfected cells with both green fluorescence and cytopathic effect (CPE) was used to inoculate into a 70-80% Vero cell monolayer at 37°C for 36 h. Subsequently, the plaque purification of the rescuing virus was performed under the condition of 2% low melting point agarose and was detected by PCR with gE-US7-9 primers (**Supplementary Table 1**). The resultant virus DNA was treated with Cre recombinase (New England Biolabs, USA) at 37°C for 30 min to remove the EGFP gene and the genome was then extracted and transfected into Vero cells as above described. After another five rounds of plaque purification, the pure recombinant PRV with the *Cap* fragment of the PCV2 genome was generated and its replication ability was determined by a 50% tissue culture infectious dose (TCID_50_).

### Immunoelectron microscopy

The cell debris in the viral supernatant was removed by a 0.22 μM filter and then a 20% Sorbitol Cushion (20% Sorbitol, 50 mM Tris HCl, 1 mM MgCl_2_, PH7.2) was used for ultra-fast centrifugation at 100000 × *g*, at 4°C for 2 h to purify the virions. Each tube was covered with the sterilized PBS and deposited at 4°C overnight. The purified virus particles were incubated in a wet box and deposited on the surface of the copper mesh. Afterward, the mesh was incubated in closed buffer diluted Cap mAbs at 37°C for 1 h, and was then transferred to a wash buffer and washed five times. Subsequently, the mesh was incubated in colloidal gold conjugated secondary antibody for 1 h and was washed with buffer five times. Finally, each mesh was cleaned in distilled water three times. The dried mesh was used to observe virion morphology under transmission electron microscope (TEM) (HT7700, Hitachi, Japan) at 80 kV and photographed with a Gatan 830 CCD camera (55).

### Immunofluorescence assay

For the immunofluorescence assays (IFA), cells were fixed with PBS (pH 7.4) containing 4% paraformaldehyde for 20 min at room temperature. After washing three times with PBST and blocking with 5% skim milk at 37°C for 1 h, the cells were incubated with the indicated primary antibodies at 37°C for 1.5 h, followed by incubation with FITC/AlexaFluor 546 labeled goat anti-rabbit or mouse IgG (KPL, America) at 37°C for another 1 h, and the nuclei were labeled with 1:5000 diluted DAPI (Solarbio, China) for 10 min. All IFA observations were performed under a fluorescence microscope (Olympas, Japan).

### Western blotting

For Western blotting (WB), cells or viral concentrate were lysed in radioimmunoprecipitation assay strong lysis buffer containing 5% SDS (Beyotime, China), 1% Triton-100 (Sigma, America), and 50 mM Tris-HCl (Aladdin, China) at pH 7.5, and analyzed on SDS polyacrylamide gel electrophoresis. The separated protein bands were transferred to a nitrocellulose blotting membrane (GE Healthcare Life Science, America). After blocking with 5% skimmed milk containing 0.1% Tween 20 (Amresco, America) at 37°C for 30 min, the cells were incubated with indicated primary antibodies overnight at 4°C. The cells were then incubated with horseradish peroxidase-conjugated anti-mouse/rabbit IgG (Kirkegaard & Perry Laboratories, America) diluted 1:4000 in 5% skimmed milk for 2 h at room temperature, and visualized with a Super Signal West Femto substrate test kit (Thermo Fisher Scientific, America) using an AI680 Image 680 (GE Health Care, America).

### Mice experiments

All animal care and experimental procedures were performed in accordance with the Animal Research Committee guidelines of Zhejiang University (No. ZJU20230315). One hundred and eighty 6-week-old specific-pathogen-free (SPF) C57BL/6 female mice were randomly divided into four groups that were intramuscularly immunized with PRV-Cap (10^7.5^ TCID_50_/ml, 0.1 ml; n=60), PRV HD/c virus (10^7.5^ TCID_50_/ml, 0.1 ml; n=25), PCV2 commercial vaccine (ZJ/C strain, 0.2 ml; n=25), or DMEM (Control, 0.1 ml; n=70), respectively. After that, the blood of five mice labeled in each group was collected from the suborbital venous plexus at 7 days post-vaccination (dpv) for Cap and gB antibody detection by ELISA and IFA assays. Four mice per group were anesthetized and euthanized at 21 dpv. Diluted serum samples were co-incubated with 100 TCID_50_ PRV-DX or PCV2 ZJ/c at 37°C for 2 h and were then inoculated with PK-15 cells for 48 h to determine serum neutralization antibody titer by IFA. Immunized mice were intraperitoneally either inoculated with a lethal dose of PRV strain DX (10^6.5^ TCID_50_/ml, 0.1 ml) or PCV2 strain ZJ/c (10^7.0^ TCID_50_/ml, 0.45ml) at 21 dpv. The clinical signs of the inoculated mice were recorded every 12 h until 14 days post PRV infection or 21 days post PCV2 challenge. The dead or euthanized mice were immediately dissected, and the brain, lung, liver, spleen, and inguinal lymph nodes were collected for histopathology, immunohistology, virus isolation, and viral load detection as described previously (56, 57). The other mice received a booster immunization at 28 days post initial immunization. The spleens collected from five mice per group at 7, 14, 21, and 28 days post initial immunization and 7 days post booster immunization were analyzed by enzyme linked immunospot (ELISpot) and flow cytometry (FCM) assays.

### Histology and immunohistology staining

Tissue samples were fixed with 10% neutrally formalin and cut into 4 mm sections after paraffin-embedded. Immunohistochemical (IHC) staining were performed afterwards to determine the presence of PRV or PCV2 antigens in tissues. Tissue sections were incubated at 37°C with 500-fold diluted PRV gC or PCV2 Cap mAbs for 1 h and then at 37°C with HRP labeled goat anti-mouse antibodies for 1 h. The freshly prepared diaminobenzidine (DAB) was displayed at room temperature to produce brown-yellow positive particle precipitation. All slides were scanned and read using a panoramic microtome scanner (Pannoramic 1000, 3DHISTECH Ltd.) and evaluated by a single veterinary pathologist blinded to the immunization groups.

### Real time quantitative PCR

The virus supernatant or tissue homogenate supernatant to be extracted was added with 20 μl protease K and 5 μl RNAase. F, bathed in water at 55°C for 2 h, and the DNA extraction phenol reagent (Solarbio, China) was added in the same volume. The reagent was vigorously mixed and allowed to stand, and centrifuged at 3000 × *g* for 10 min. The supernatant was added to equal volume premixed chloroform: isoamyl alcohol (24:1) and mixed gently. After centrifugation at 3000 × *g* for 10 min, the supernatant was added into 2-2.5 times the volume of pre-cooled anhydrous ethanol and treated at -20°C for 30 min. After centrifugation at 14000 × *g* for 10 min, the supernatant was discarded and the precipitation was washed with 1 ml 75% ethanol. The precipitation was completely dissolved with TE buffer after drying, and PRV gB, PCV2 Rep, and gapdh mRNA were then detected using ChamQ Universal SYBR qPCR Master Mix (Vazyme Biotechnology; Q711-02). Primers of gB and Rep are shown in **Supplementary Table 1**.

### ELISpot assay

An ELISpot assay was conducted as stated (58). Briefly, single spleen cell samples from 2-week-immunized mice euthanized by CO_2_ were prepared with RPMI 1640 (Gibco, America) containing 2% FBS through 200-mesh cell filtration screens. The cells were then treated with ammonium chloride potassium (ACK) lysis buffer (Solarbio, China) at 4°C for 5 min and then terminated with RPMI 1640 supplemented with 2% FBS in the same volume. After 500 × *g* centrifugation, the precipitate was suspended in RAPI 1640 containing 10% FBS. For the ELISpot assay, 5×10^5^ cells/well were placed on ELISPOT PVDF 96-well plates (U-CyTech, Netherlands) pre-coated with mouse IFN-γ antibody and inactivated PCV2-ZJ/c or PRV-DX (MOI=1) as antigen or PMA/Ionomycin (MCE, America) mixture as positive control were added at 37°C for 24 h. Then, the ELISpot plates were treated according to the instructions from the manufacturer. The spots stained with 3-Amino-9-Ethylcarbazole (AEC) dye were quantified by the automatic immunospot analyzer (Cellular Technology Ltd., America) to determine the cytokine secretion amount of spleen cells in each group.

### FCM assay

Splenocyte suspensions from immunized mice were prepared according to the ELISpot assay. For B cell surface staining, 1 × 10^6^ cells plated in 96-well V-bottom plates were pretreated with Mouse Fc block (BD Biosciences, America) at a concentration of 25 μg/ml at 4°C for 10 min. Subsequently, the resultant cells were stained with the surface antibodies diluted with cell PBS containing 2% BSA and 25 μg/ml Fc block at 4°C for 30 min. For endonuclear staining, 1 × 10^6^ cells were incubated with the surface antibodies at 4°C for 30 min. After centrifuge, the cells were treated with the Transcription Factor Buffer Set (BD Biosciences, America) for cell fixation and permeabilization. Cells were subsequently incubated with intranuclear antibodies diluted in permeabilization buffer at 4°C for 1 h. For intracellular cytokine staining (ICS), 2 × 10^6^ splenocyte cells plated in 96-well U-bottom plates were incubated with corresponding viral antigen for stimulation at 37°C for 4 h. The resultant cells were incubated with Brefeldin A (MCE, America) with a concentration of 5 µg/ml overnight and were fixed with 4% PFA on an ice bath for 5 min and then permeabilizated with Cytofix/Cytoperm Kit (BD Biosciences, America) for 20 min (48, 59). Subsequently, the cells were incubated with mouse anti-IFN-γ, TNF-α, and IL-2 antibodies at 4°C for 30 min, washed twice, and resuspended in 200µl cell PBS containing 0.5% paraformaldehyde (PFA) for FACS analysis. At least 2×10^5^ cells were collected using a BD FACSVerse Fortessa (BD Biosciences, America), and the data were analyzed using FlowJo software (Tree Star Inc., America). Details of antibodies used in the FCM are listed in **Supplementary Table 2**.

### Statistical analysis

Statistical analysis was performed using GraphPad Prism 8.0 software, and all data are presented as the mean ± SD of three independent experiments. For all experiments, *p* < 0.05 was considered statistically significant. In the figures, not significant (ns): *p* > 0.05; *, *p* < 0.05; **, *p* < 0.01; ***, *p* < 0.001.

## Results

### Construction of the recombinant PRV type II with PCV2 Cap protein

To construct a recombinant virus expressing the Cap protein of PCV2 on the PRV II envelope, 293T cells were co-transfected with PRV II HD/c genome, pUC18-Cap-EGFP transfer vector, and US9 CRISPR-Cas9 gene editing vector to generate a recombinant PRV expressing both the Cap of PCV2 and EGFP (PRV-Cap-EGFP). At 48 h post transfection, the cells with an obvious green fluorescence were selected to inoculate into the Vero cell monolayer. To further generate PRV-Cap without the EGFP label, 293T cells were transfected with the Cre recombinase-treated PRV-Cap-EGFP genome. Subsequently, the cells with CPE and without fluorescence were used to infect Vero cells to obtain PRV-Cap. In immunofluorescence analysis, EGFP, PCV2 Cap, and VP5 of PRV were detected in PRV-Cap-EGFP infected cells, and only Cap and VP5 proteins were detectable in PRV-Cap infected cells (**Figure 1B**). Similar results are shown in the Western blot and PCR analyses (**Figures 1C, D**). These data indicate that the *Cap* gene of PCV2 was successfully inserted into the PRV genome and the PRV-Cap was rescued.

### Characteristics of chimeric PRV with Cap protein of PCV2

**Figure 2:**
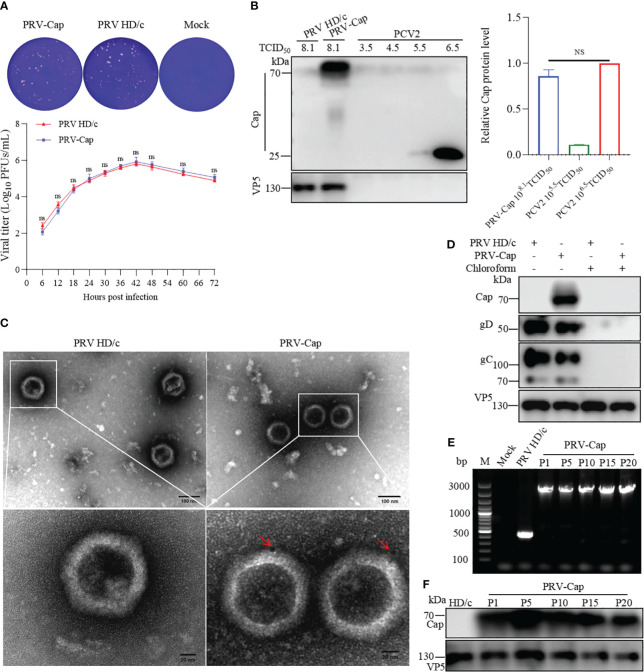
Detection of expression and genetic stability of Cap-gE chimeric protein in PRV-Cap. **(A)** One-step growth curves and plaque detection of PRV-Cap and parent PRV HD/c virus (MOI=1) infected PK-15 cells. The infected cells were incubated at 37°C for 2 h, washed with DMEM three times, and cells were finally cultured with DMEM containing 2% FBS. Viral titers were measured at indicated time points post infection. The plaque formation unit (PFU) was detected by the limited dilution plaque assay and TCID_50_ was determined by Reed-Muench method. **(B)** The abundance of the Cap-gE fusion protein in the supernatant of PRV-Cap was detected by Western blotting compared with the original Cap protein of PCV2. **(C)** The virus particles purified by 20% Sorbitol Cushion ultra-centrifugation were examined by transmission electron microscopy with anti-Cap mAbs as described in Materials and methods. **(D)** The purified Cap virions were violently mixed with chloroform and ice bath for 20 min to remove the virus envelope for Western blotting detection. **(E)** The genome of PRV-Cap was extracted, and PCR detection and sequencing were performed with gE-US7-9 primers. **(F)** Western blotting detection of each generation of 10^8.0^ TCID_50_/ml PRV-Cap supernatant. NS, p >0.05.

To analyze the replication capacity of recombinant chimeric PRV-Cap, the virus titer was detected by a one-step growth curve using the plaque technique. The plaque formation test showed that the virus titer of the PRV-Cap had no significant difference compared to its parent PRV HD/c virus (**Figure 2A**), suggesting that the insertion of the *Cap* fragment in the gene *gE* does not affect the ability of PRV replication. To detect the expression level of Cap protein in PRV-Cap, we conducted a comparative analysis of PCV2 and PRV-Cap viruses. A Western blot assay revealed that the Cap concentration of the PRV-Cap with 10^8.1^ TCID_50_/ml had no significant difference to PCV2 with 10^6.5^ TCID_50_/ml (**Figure 2B**). To identify whether the Cap protein was orientated in the envelope of PRV II virion, the ultracentrifuged and purified PRV-Cap virions were stained with a colloidal gold conjugated anti-Cap antibody and observed by TEM. The result indicated that the Cap protein was displayed on the envelope of the PRV virion (**Figure 2C**). Consistently, the Cap protein was undetectable in the purified PRV-Cap with chloroform treatment in Western blot analysis (**Figure 2D**), revealing that the Cap protein of PCV2 was located on the PRV envelope. Moreover, to test the genetic stability of the *Cap* gene in recombinant PRV, the PRV-Cap was continuously passaged in PK-15 cells 20 times, and the genome of each generation was extracted for PCR detection. The results showed that the protein and nucleotide fragment of the *Cap* gene in the PRV-Cap could be detected from the 1^st^ passage to 20^th^ passage, revealing that the *Cap* gene in PRV-Cap was stable (**Figures 2E, F**). Collectively, these data confirmed that the PRV-Cap is a recombinant chimeric virus and that the Cap protein of PCV2 is embedded in the envelope of PRV.

### Immunogenicity and safety of the PRV-Cap

**Figure 3:**
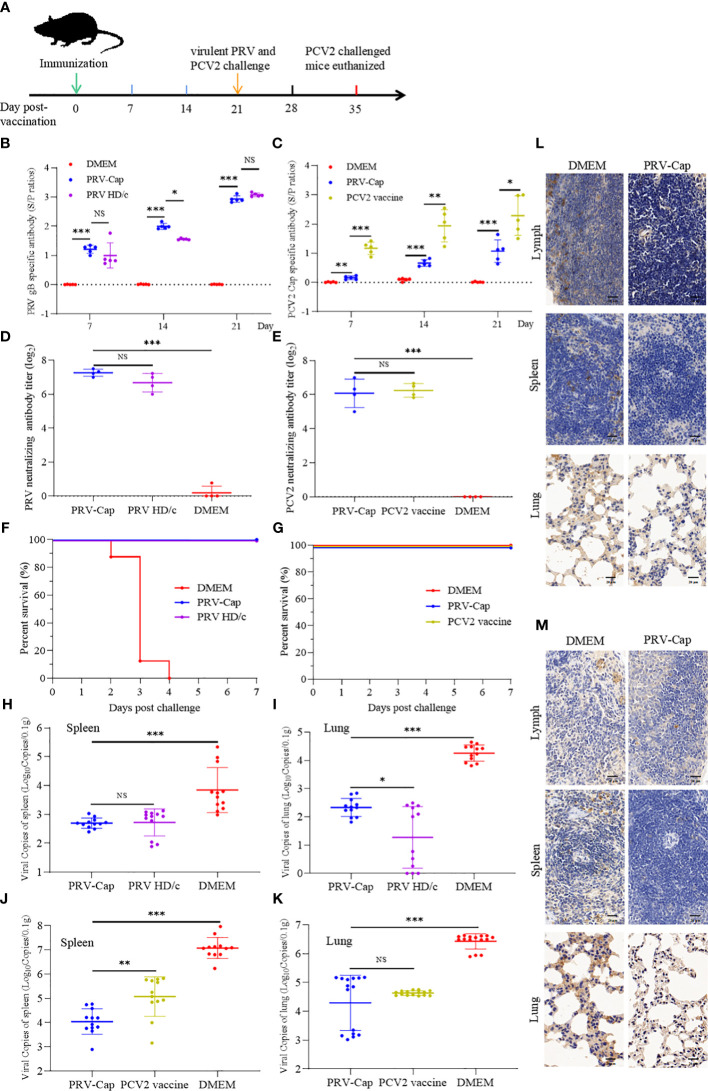
Immunogenicity of the PRV-Cap. **(A)** The mouse model of PRV-Cap vaccination. **(B, C)** The gB and Cap ELISA antibody titers at 7, 14, and 21 dpv. **(D, E)** Neutralization antibody titers against PRV or PCV2 at 21 dpv. **(F, G)** Survival rates of mice immunized post 3 weeks against PRV-DX (10^6.5^ TCID_50_/ml, 0.1 ml) or PCV2 ZJ/c (10^7.0^ TCID_50_/ml, 0.45ml) challenge. **(H, I)** The viral loads in the lungs and spleens of mice were measured by qPCR assay on 3^rd^ day after PRV-DX challenge, and PRV genomic copies were calculated according to the CT value of the PRV-gB standard curve. **(J, K)** The viral loads in the lungs and spleens of mice were measured by qPCR assay on day 21 after PCV2 challenge, and PCV2 genomic copies were calculated according to the CT value of the PCV2-Rep standard curve. **(L, M)** Immunohistochemical analysis of the lungs, spleens and inguinal lymph nodes of PRV DX or PCV2 ZJ/c challenged mice. Magnification, 80×; Scale bars, 20 μm. Data are expressed as means ± standard errors of the means. NS, *p* >0.05; *, *p* < 0.05; **, *p* < 0.01; ***, *p* < 0.001.

To detect the immunoprotective ability of the PRV-Cap to host, the PRV-Cap was inoculated into 6-week-old C57BL/6 mice, while the parent PRV HD/c virus, PCV2 commercial ZJ/C inactivated vaccine, and DMEM were used as controls. The strategies and detailed time points of mice experiments are shown in **Figure 3A**. We detected antibody dynamics to evaluate the humoral immune response of the PRV-Cap inoculated mice. ELISA assays showed that the antibodies to both gB and Cap were detectable in the PRV-Cap inoculated mice at 7 dpv and reached a peak at 21 dpv. Interestingly, in PRV-Cap inoculated mice, the anti-gB antibodies were slightly higher than in parent PRV HD/c virus immunized mice (**Figure 3B**). Conversely, the antibodies against the Cap were significantly lower in PRV-Cap inoculated mice than in ZJ/C vaccine-immunized mice (**Figure 3C**). However, a similar trend was shown for neutralizing antibodies against PRV at 21 dpv (**Figures 3D, E**). These data suggest that the Cap protein in the PRV-Cap has a synergistic promoting effect on humoral immune responses of PRV. At 21 days post-inoculation, the PRV-Cap inoculated mice challenged with PCV2 and lethal PRV had a 100% survival, similar to those immunized with their parent PRV HD/c virus and ZJ/C vaccines (**Figures 3F, G**). The above-mentioned data verify that the PRV-Cap inoculated mice had complete immune resistance to PCV2 induced infection and death caused by lethal PRV infection.

To evaluate the environment safety of PRV-Cap, and replication of pathogenic PRV and PCV2, we conducted PCR detection, virus isolation, and histological and immunohistological observations of the PRV-Cap immunized mice. The virus excretion detection revealed that the fragments of the *gE* and *gB* genes were undetectable in PCR and the PRV-Cap could not be isolated from fecal samples of mice within 1 week after inoculation (data not shown). This suggests that mice immunized with the PRV-Cap do not excrete viruses into the environment through the digestive tract and are environmentally safe. After being challenged with PCV2 and lethal PRV, the spleens and lungs of the PRV-Cap immunized mice were observed to have no PRV and PCV2 antigens in immunohistological staining, in comparison with mock immunized mice (**Figures 3L, M**). Concurrently, in the qPCR assay, virus copies in spleens and lungs of the PRV-Cap immunized mice were significantly lower than that of mock immunized mice after PCV2 and PRV challenges (**Figures 3H–K** and **Supplementary Figure 1**). Moreover, in the virus isolation assay, PCV2 and PRV were undetectable in the spleens and lungs of the PRV-Cap immunized mice with PRV and PCV2 challenges for three consecutive rounds in PK-15 cells. These results demonstrate that the immunized mice of PRV with the chimeric Cap protein were environmentally safe.

### PRV-Cap immunization induced early activation and expansion of lymphocytes

**Figure 4:**
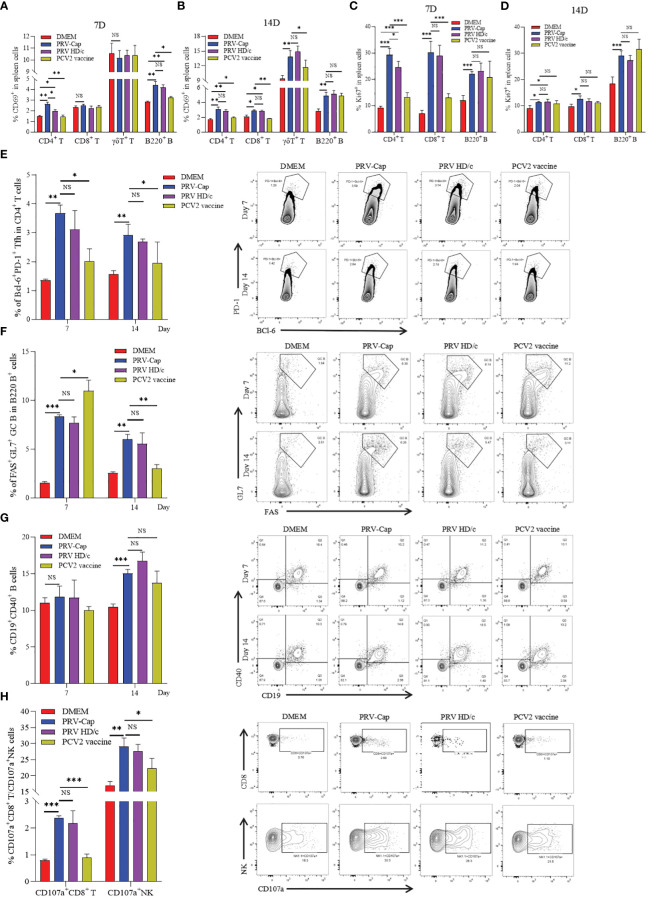
Dynamic changes of activation and expansion of different immune cell subsets after PRV-Cap immunization. **(A, B)** The expression of activation marker CD69 in CD4 T cells, CD8 T cells, γδ T cells, and B cells at 7 and 14 dpv. **(C, D)** The expression of expansion marker Ki67 in CD4 T cells, CD8 T cells, and B cells at 7 and 14 dpv. **(E, F)** The proportion and representative cytometric profiles of Tfh cells (CD4^+^PD-1^+^BCl-6^+^) and GC B cells (B220^+^GL7^+^FAS^+^) at 7 and 14 dpv. **(G)** The percentage and representative cytometric profiles of CD40-positive B cells (CD19^+^CD40^+^) at 7 and 14 dpv. **(H)** The expression and representative cytometric profiles of CD107a in NK cells and CTLs at 14 dpv. NS, *p* >0.05; *, *p* < 0.05; **, *p* < 0.01; ***, *p* < 0.001.

Type II C-type lectin receptor CD69 is a classical early marker of lymphocyte activation (60, 61), and nuclear antigen Ki67 is widely used to trace the proliferative activity of T cells after vaccine immunization or pathogen infection (62–64). To detect the activation and expansion of cellular and humoral immunity induced by PRV-Cap, we used multiparameter FCM to analyze the dynamics of CD69 and Ki67 in different T cell subsets and B cells. Based on the gating strategy for multicolor shown in **Supplementary Figures 2A, B**, in FCM analysis, it was observed that CD69^+^CD4^+^ T cells and CD69^+^B220^+^ B cells were significantly activated in Cap- and HD/c- virus vaccinated mice on the 7^th^ day, and significant upregulation of CD69^+^CD8^+^ T cells and CD69^+^γδ T cells did not appear until the 14^th^ day (**Figures 4A, B**). Moreover, Ki67^+^CD4^+^ T cells, Ki67^+^CD8^+^ T cells, and Ki67^+^B220^+^ B cells all showed a significant increase on the 7^th^ day, and Ki67 levels of CD4^+^ T cells and CD8^+^ T cells declined on the 14^th^ day while B cells remained unchanged (**Figures 4C, D**). Noticeably, the number of CD69^+^CD4^+^ T cells and Ki67^+^CD4^+^ T cells in PRV-Cap immunized mice was significantly higher than that in parent PRV HD/c virus immunized mice on the 7^th^ day. Next, we analyzed the percentage of Tfh cells (CD4^+^PD-1^+^BCl-6^+^), GC B cells (B220^+^GL7^+^FAS^+^), and CD40-positive B cells (CD19^+^CD40^+^) in the spleens of immunized mice on the 7^th^ and 14^th^ day, according to the gating strategy shown in **Supplementary Figures 2B, C**. **Figures 4E–G** revealed that both PRV-Cap and PRV HD/c virus inoculated mice had a significant increase of Tfh, GC B, and CD40 positive B cells without a difference. In addition, the activation of NK cells and CTLs was analyzed using a lysosomal-associated membrane protein marker CD107a (65, 66) according to the gating strategy shown in **Supplementary Figure 2D**. **Figure 4H** shows that CD107a^+^ NK cells and CD107a^+^CD8^+^ CTLs were significantly upregulated after PRV-Cap or PRV HD/c virus inoculation. Generally, the above-mentioned data demonstrate that the early activation and expansion of lymphocytes induced by PRV-Cap was superior to the parent PRV HD/c virus.

### PRV-Cap immunization induced Th1 cytokine specific for PRV and PCV2

**Figure 5:**
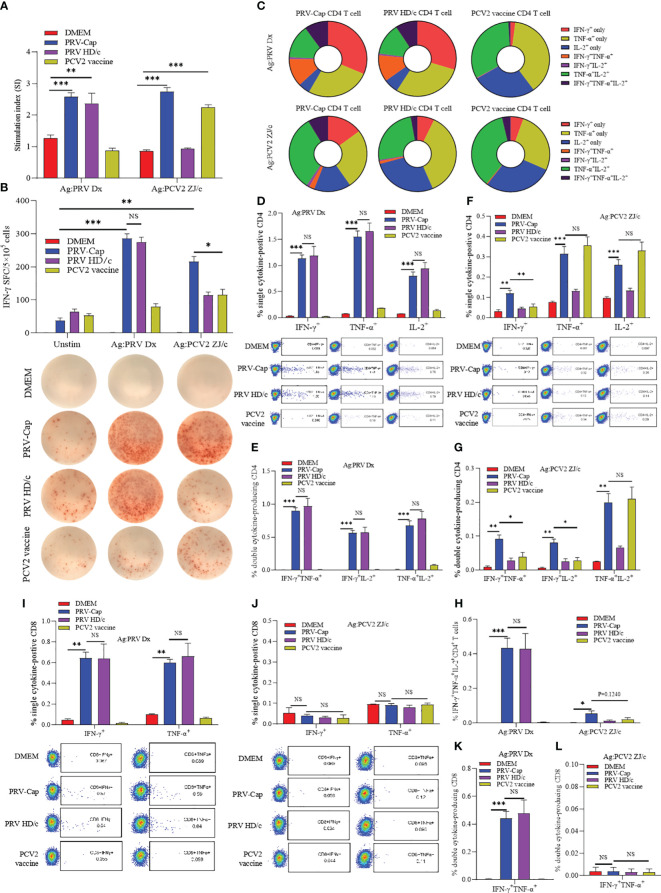
PRV and PCV2 specific cytokine production profile from splenocytes of PRV-Cap vaccinated mice at 14 dpv. **(A)** The specific proliferation response of splenocytes was detected using the CCK8 method. Splenic lymphocyte suspension was stimulated with PRV-DX or PCV2 inactivated antigen (MOI=1) for 48 h, then antigen-specific proliferative response was detected at OD450 using CCK8. The stimulation index (SI) = (test group OD450 - blank control OD450)/(negative control OD450 - blank control OD450). **(B)** Representative images of IFN-γ ELISpot wells and mean spot size from the various vaccine groups. The secreting spots forming cells (SFC) were counted using the ELISpot method after 24 h stimulation with PRV-DX or PCV2 inactivated antigen. **(C)** The proportion of PRV or PCV2 specific CD4^+^ T cells that produced IFN-γ, TNF-α or IL-2 single cytokine, any two cytokines, and triple cytokines. **(D–H)** Percentage of CD4^+^ T cells that produced single, double, and triple representative TH1 cytokines after PRV-DX or PCV2 stimulation (MOI=1, 24h). **(I–L)** Percentage of CD8^+^ T cells that produced single or double cytokines after PRV-DX or PCV2 stimulation (MOI=1, 24h). Data expressed as mean ± sd from five mice per group. NS, *p* >0.05; *, *p* < 0.05; **, *p* < 0.01; ***, *p* < 0.001.

To detect the PRV-Cap-induced specific cellular immune response, splenocytes from the immunized mice at 14 days post-immunization were stimulated with either inactivated PRV-DX or PCV2. As shown in **Figure 5A**, the PRV-Cap vaccination induced dual (PRV and PCV2)-specific lymphocyte expansion, whereas the PRV HD/c virus or PCV2 vaccine immunization only induced a single specific expansion upon ex vivo re-stimulation. An ELISPOT assay showed that the vaccination of PRV-Cap induced more PCV2-specific IFN-γ spot-forming cells (SFC) than PCV2 immunization but induced comparable number of PRV-specific IFN-γ SFC to PRV HD/c virus immunization (**Figure 5B**). The gating strategy for cytokine detection is shown in **Supplementary Figure 3A**. In FCM assay (**Figures 5C–H**), PRV-specific single cytokine (IFN-γ^+^, TNF-α^+^ or IL-2^+^), double cytokine (IFN-γ^+^TNF-α^+^, IFN-γ^+^IL-2^+^ or TNF-α^+^IL-2^+^), and triple cytokine (IFN-γ^+^TNF-α^+^IL-2^+^) expressing CD4 T cells showed a significant increase in PRV-Cap and PRV HD/c virus immunized mice upon ex vivo restimulation with inactivated PRV compared to mock immunized mice. Interestingly, the PRV-Cap immunized mice produced more PCV2-specific IFN-γ secreting CD4 T cells than the PCV2 vaccine immunized mice upon ex vivo restimulation with inactivated PCV2. Similarly, Cap or PRV HD/c virus immunization induced PRV-specific IFN-γ^+^, TNF-α^+^, or IFN-γ^+^TNF-α^+^ CD8^+^ T cells, but there was no significant difference between PRV-Cap and PRV HD/c virus. No CD8 T cells expressed with PCV2-specific cytokines were detected in the PRV-Cap and PCV2 vaccine immunized mice (**Figures 5I–L**). These data indicate that PRV-Cap immunization activates CD4^+^ and CD8^+^ T cell subsets, eliciting better T-cell immunity than PCV2 but not compromising anti-PRV immunity.

### PRV-Cap immunization produced specific effector memory T and B cells

**Figure 6:**
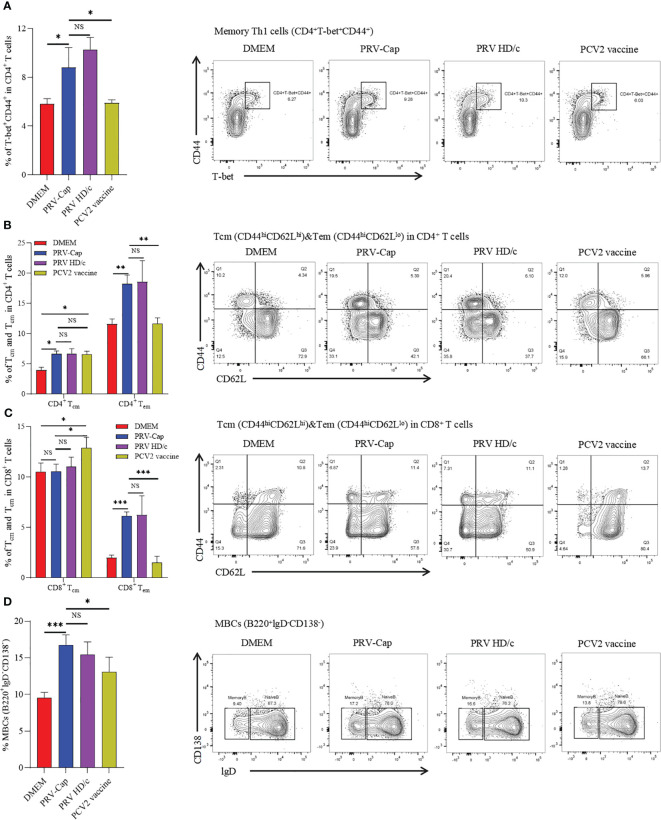
Percentage of memory T and memory B cells in splenocytes at 14 dpv. **(A)** The proportion and representative cytometric diagrams of CD4^+^ memory Th1 cells. **(B, C)** Statistical analysis and representative cytometry diagrams of Tcm and Tem in CD4^+^ or CD8^+^ T cells. **(D)** The proportion and representative cytometric diagrams of MBCs in B220 positive B cells. Data expressed as mean ± sd from five mice per group. NS, *p* >0.05; *, *p* < 0.05; **, *p* < 0.01; ***, *p* < 0.001.

Memory T cells (Tm) and memory B cells (MBCs) are activated and differentiated during secondary infection, producing a fast and powerful immune response, which is particularly important for resistance to viral infection (24, 67–69). Therefore, we measured CD4^+^ and CD8^+^ memory T cells in primarily immunized mice at 14 days post-immunization according to the gating strategy shown in **Supplementary Figure 3B**. In this study, it was observed that memory Th1 cells (CD4^+^T-bet^+^CD44^+^) showed a significant increase in mice inoculated with PRV-Cap and PRV HD/c virus compared to mice immunized with a mock treatment or PCV2 commercial vaccine. There was no significant difference between the PRV-Cap and PRV HD/c viruses (**Figure 6A**). In Tm, two cell subsets of central memory T cells (Tcm) and effector memory T cells (Tem), were defined based on differential expression of lymphocyte homing marker CD62L. Therefore, we measured the percentage of Tcm (CD44^hi^CD62L^hi^) and Tem (CD44^hi^CD62L^lo^) in CD4^+^ Tm and CD8^+^ Tm. The results showed that PRV-Cap and PRV HD/c virus immunization induced a significant increase in CD4^+^ Tcm, CD4^+^ Tem, and CD8^+^ Tem in mice, while the commercial PCV2 vaccine only showed a significant increase in CD4^+^ Tcm and CD8^+^ Tcm (**Figures 6B, C**). In addition, we measured the percentage of MBCs (B220^+^lgD^-^CD138^-^) based on the gating strategy shown in **Supplementary Figure 2C**. **Figure 6D** shows that PRV-Cap-induced MBCs are higher than PRV HD/c virus and PCV2 at 7 days post-immunization. These data demonstrate that PRV-Cap immunization induces the production of CD4^+^ and CD8^+^ effector memory T cells.

### The booster immunization with PRV and PCV2 recalls the reactivation of IFN-γ^+^IL-2^+^CD4^+^ and IFN-γ^+^TNF-α^+^CD8^+^ T cells

**Figure 7:**
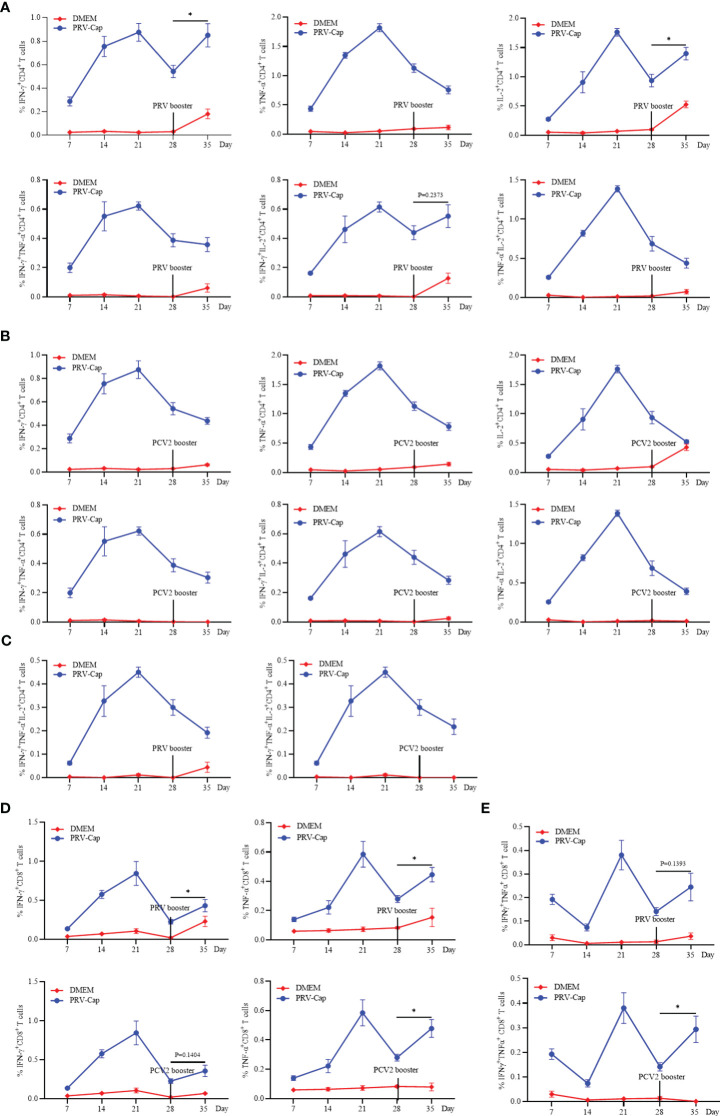
Analysis of the memory response of PRV-Cap induced T cells. The booster immunization with PRV-DX (10^6.5^ TCID_50_/ml, 0.1 ml) or PCV2 (10^7.0^ TCID_50_/ml, 0.45ml) was performed in mice at 28 days after initial immunization with the PRV-Cap. **(A–C)** Percentage of CD4^+^ T cells stimulated by PRV-Cap (MOI=1, 24h) to produce single, double, and triple representative TH1 cytokines at 7, 14, 21, and 28 dpv and at 7 days post booster immunization with PRV-DX or PCV2. **(D, E)** Percentage of CD8^+^ T cells stimulated by PRV-Cap (MOI=1, 24h) to produced single or double cytokines at 7, 14, 21, and 28 dpv and 7 days post booster immunization with PRV-DX or PCV2. Data expressed as mean ± sd from five mice per group. NS, *p* >0.05; *, *p* < 0.05; **, *p* < 0.01; ***, *p* < 0.001.

To further evaluate the memory response of the PRV-Cap induced T cells, the booster immunization with PRV-DX, PCV2-ZJ/c, and PRV-Cap was performed in mice at 28 days post initial immunization with the PRV-Cap. Subsequently, cytokine expression in T cells was examined by FCM at 7 days post-booster immunization. **Figures 7A, C** showed that the proportions of PRV-specific IFN-γ^+^CD4^+^ and IL-2^+^CD4^+^ T cells increased significantly after PRV booster immunization, while the percentage of TNF-α^+^CD4^+^, IFN-γ^+^TNF-α^+^CD4^+^, TNF-α^+^IL-2^+^CD4^+^, and IFN-γ^+^TNF-α^+^IL-2^+^CD4^+^ T cells continued to decrease, demonstrating that the PRV booster immunization only resulted in the upregulation of IFN-γ^+^IL-2^+^CD4^+^ T cells. Conversely, the percentage of PCV2-specific single cytokine, double cytokine, and triple cytokine expressing CD4 T cells all showed a decrease in mice with PCV2 booster vaccination (**Figures 7B, C**), indicating that the PCV2 booster immunization could not stimulate an obvious reactivation of CD4 T cells. Similar trends appeared in mice with booster immunization with PRV-Cap at 28 days post initial immunization with the PRV-Cap (**Supplementary Figure 4**). Moreover, the percentage of IFN-γ^+^CD8^+^, TNF-α^+^CD8^+^, and IFN-γ^+^TNF-α^+^CD8^+^ T cells showed a significant upregulation after both PRV and PCV2 booster immunization (**Figures 7D, E**). These results proved that PRV booster vaccination, rather than PCV2, could cause the memory response of IFN-γ^+^IL-2^+^CD4^+^ T cells and the activation of IFN-γ^+^TNF-α^+^CD8^+^ T cells could be reactivated after booster vaccinations.

## Discussion

In recent years, the generation of recombinant vector vaccines has become an important research focus (70, 71). As an important zoonotic virus, PRV can take advantage of its large genome and multiple non-essential genes to construct recombinant attenuated vaccines carrying foreign genes. Research has shown that the PRV SA215 strain (TK^-^/gE^-^/gI^-^ deleted) can be employed as a vector to insert PPV VP2 and EGFP expression box into the gI site using homologous recombination technology (18). Furthermore, gI and gE were replaced by the SVA VP2 and EGPF to obtain recombinant PRV (19). However, a major problem is that the foreign genes in recombinant PRV are only integrated into the genome for independent expression, rather than assembled on the surface of the virion. In this study, we selected a gE/TK virulence gene deficient PRV HD/c virus as the recombinant virus skeleton. We inserted the Cap gene of PCV2 into the extracellular domain of the PRV *gE* gene and added a cytomegalovirus promoter in front of the fusion gene to enhance the expression of the inserted gene. We also verified that the Cap protein expressed was embedded on the surface of the PRV envelope. In animal immunization tests, the recombinant PRV with the *Cap* gene induced higher levels of anti-PRV neutralizing antibodies compared to the parent PRV HD/c virus strain and exhibited excellent immune protection against both PRV and PCV2. These data demonstrate for the first time that there is no immune antagonism between the PCV2 Cap protein and PRV when the recombinant PRV carrying the Cap protein is inoculated, and that the Cap protein enhances the immune efficacy of PRV. Therefore, in view of the high replication ability of PRV-Cap, this study implies that using the recombinant PRV with the *Cap* gene of PCV2 to produce the Cap protein of PCV2 can not only significantly overcome the deficiency of PCV2 replication ability, but also achieves the effect of preventing two diseases with one dose of vaccine.

The effective host defense against viral infection depends on humoral and cellular immunity (20, 72). However, the mechanism of specific immune response induced by recombinant PRV has not been discussed in detail. Considering that the introduction of the Cap protein may affect the cellular and humoral immunity of PRV, we detected the activation and/or proliferation levels of CD4 T cells, CD8 T cells, γδT cells, NK cells, and B cells. Flow cytometry analysis revealed that PRV-Cap effectively mediated the specific activation and proliferation of all the aforementioned cell subsets. Previous studies have reported that Tfh can regulate the production of high-affinity antibodies by inducing GC B maturation (23–25). In this study, we demonstrated that PRV-Cap effectively stimulates the production of Tfh, GC B, and CD40-positive B cells, with no significant differences compared to PRV HD/c virus, indicating that the insertion of the Cap protein does not significantly affect the overall immunology of PRV. It is worth mentioning that the proportion of CD69 and Ki67 positive CD4 T cells of the recombinant PRV with the Cap protein was higher than that in the parent PRV HD/c virus on the 7^th^ day, accompanied by a slight increase in the percentage of Tfh. Heterologous immunity mediated by the bystander effect in viral infection (73) may be the reason for the difference in antibody levels between PRV-Cap and PRV HD/c virus in the early immunization period.

Cytokines are small polypeptides or proteins that transmit information within cells and play a crucial role in immune regulation (74). IFN-γ, IL-2, and TNF-α are representative functional antiviral cytokines secreted by antigen-specific Th1 cell subpopulations and are essential for antiviral infection and immunological evaluation of vaccines (43, 44). IFN-γ, as the most vital antiviral cytokine, not only collaborates with TNF-α to inhibit viral replication, but also stimulates specific cytotoxic immunity and activates macrophages to phagocytic pathogens by recognizing virus-associated major histocompatibility complex (MHC) on the cell surface (45–48). Studies have shown that CD8^+^ T cells can also secrete a variety of pro-inflammatory cytokines, mainly IFN-γ and TNF-α, to inhibit viral replication, and express various chemokines to recruit inflammatory cells to the site of infection (42, 75). In this study, both PRV-Cap and parent PRV HD/c virus induced strong PRV-specific cytokine production and similar cytokine expression profiles in CD4 T cells and CD8 T cells, and PRV-Cap synchronously mediates the production of PCV2-specific cytokines, suggesting that the insertion of PCV2 Cap protein did not affect the overall immunogenicity of PRV. Notably, the cytokines induced by PCV2 inactivated vaccine were mainly IL-2 and TNF-α secreted by CD4 T cell subsets, and only a small amount of IFN-γ was produced at the early stage, while the PRV-Cap induced stronger IFN-γ production and a higher proportion of IFN-γ SC expression profile compared to the inactivated commercial PCV2 vaccine. It has been reported that PCV2 inactivated and subunit vaccines exhibit weak IFN-γ secretion (76). The addition of IFN-γ enhances the proliferative response of PCV2-specific T lymphocytes and shows a better protective effect (77). Therefore, these data suggest that the recombinant PRV with the Cap protein may induce stronger PCV2-specific cellular immunity than PCV2 inactivated vaccine.

Memory T and B cells produced by vaccination are activated and differentiated during secondary infection or booster vaccination, exerting a rapid and robust immune response that is particularly important for antiviral infection (24, 67–69). In this study, we examined the phenotypes of Tm and MBCs after immunization. The results show that both the PRV-Cap and PRV HD/c viruses significantly increased memory Th1 cells, CD4^+^ Tem, CD8^+^ Tem, and MBCs, with a slight increase in CD4^+^ Tcm, indicating that Cap insertion did not affect the overall memory cell phenotype. At the same time, our results show that inactivated commercial PCV2 exhibits weak T cell immune memory dominated by Tcm and weak B cell memory. To further evaluate the memory response of the PRV-Cap-induced T cells, we detected dynamic changes in cytokines in mice booster immunized with PRV-DX or PCV2-ZJ/c at 28 days post-immunization. PRV booster immunity only led to the upregulation of PRV-specific IFN-γ^+^IL-2^+^CD4^+^ T cells and IFN-γ^+^TNF-α^+^CD8^+^ T cells, while PCV2 booster immunization only enhanced IFN-γ^+^TNF-α^+^CD8^+^ T cells but did not significantly stimulate CD4 T cell reactivation. This difference most likely corresponds to weak CD4 T memory cells mediated by PCV2 inactivated vaccine.

In summary, we constructed a recombinant PRV carrying the *Cap* gene of PCV2. The recombinant virus stably assembled the Cap-gE fused protein on the surface of its viral envelope and induced both humoral and cellular immunity against PRV and PCV2, which indicates that the PRV-Cap is a promising candidate vaccine against both PRV and PCV2.

## Data Availability

The raw data supporting the conclusions of this article will be made available by the authors, without undue reservation.
