# Corrigendum: Immunological characteristics of a recombinant alphaherpesvirus with an envelope-embedded *Cap* protein of circovirus

**DOI:** 10.3389/fimmu.2024.1515309

**Published:** 2024-11-18

**Authors:** Chenhe Lu, Haimin Li, Wenjing Chen, Hui Li, Jiayu Ma, Peng Peng, Yan Yan, Weiren Dong, Yulan Jin, Shiyue Pan, Shaobin Shang, Jinyan Gu, Jiyong Zhou

**Affiliations:** ^1^ MOA Key Laboratory of Animal Virology, Zhejiang University Center for Veterinary Sciences, Hangzhou, China; ^2^ College of Veterinary Medicine, Yangzhou University, Yangzhou, China; ^3^ State Key Laboratory for Diagnosis and Treatment of Infectious Diseases, First Affiliated Hospital, Zhejiang University, Hangzhou, China

**Keywords:** chimeric pseudorabies virus, circovirus, immunity, memory responses, IFN-γ

In the published article, there was an error. In the **Materials and Methods** section, we conducted Cas9 editing on the homologous right arm region, US9 and US2, and the final site was US2 instead of US9.

A correction has been made to **Materials and Methods**, *Construction for Cap protein of PCV2 transfer and US9 CRISPR-Cas9 gene editing vectors*. This section sub-heading previously stated: “Construction for Cap protein of PCV2 transfer and US9 CRISPR-Cas9 gene editing vectors”.

The corrected sub-heading appears below:

“Construction for Cap protein of PCV2 transfer and US2 CRISPR-Cas9 gene editing vectors”.

In the published article, there was an error in **Figure 1A** as published. As stated above, we conducted Cas9 editing on the homologous right arm region, US9 and US2, and the final site was US2 instead of US9. The corrected **Figure 1A** and its caption appear below.

**Figure 1:**
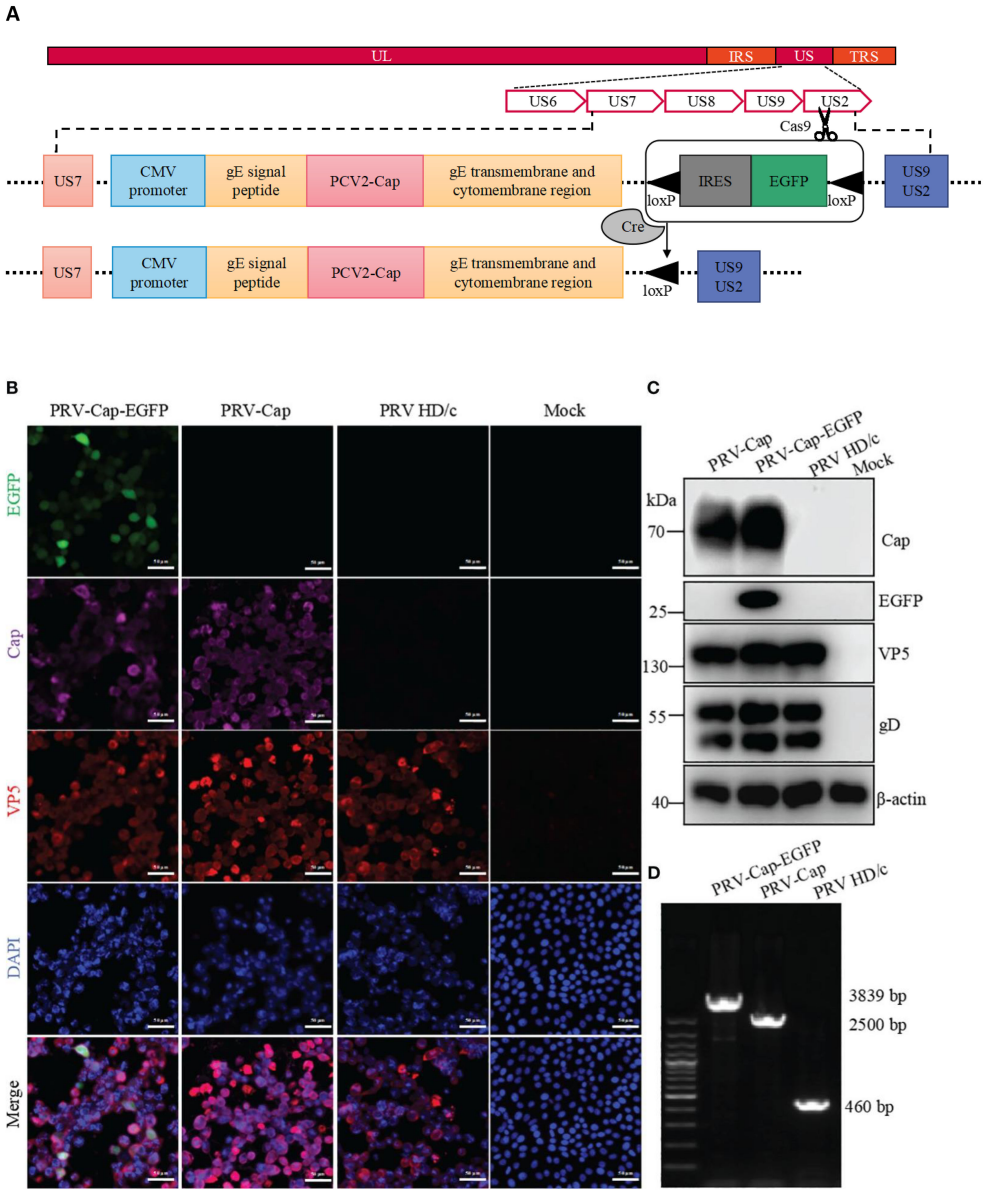
Generation of recombinant PRV with the Cap protein of PCV2. **(A)** The construction strategy of PRV-Cap. The PCV2 *Cap* gene was inserted into the *gE* extracellular region of parent PRV HD/c virus strain by homologous recombinant transfer vector, and then the fluorescent labeled *EGFP* gene was removed *in vitro* by the Cre-LoxP recombinant enzyme system to obtain the recombinant PRV with only the exogenous Cap gene. **(B)** IFA and **(C)** Western blotting assays of Cap-gE fusion protein expression in Vero cells inoculated with 1 MOI of PRV-Cap-EGFP, PRV-Cap, and PRV HD/c virus at 24 h post infection **(D)**Identification of inserting the *Cap* gene in Cap-EGFP and PRV-Cap by nucleic acid electrophoresis using gE-US7-9 primers.

The authors apologize for these errors and state that they do not change the scientific conclusions of the article in any way. The original article has been updated.

