# Supplementary material for: Immunological characteristics of a recombinant alphaherpesvirus with an envelope-embedded Cap protein of circovirus
Source: Front Immunol. 2024 Jul 16;15:1438371. doi: 10.3389/fimmu.2024.1438371 (PMC11286414; doi:10.3389/fimmu.2024.1438371)
Supplement: Supplementary file 1 [file Presentation1.pptx]

## Slide 1
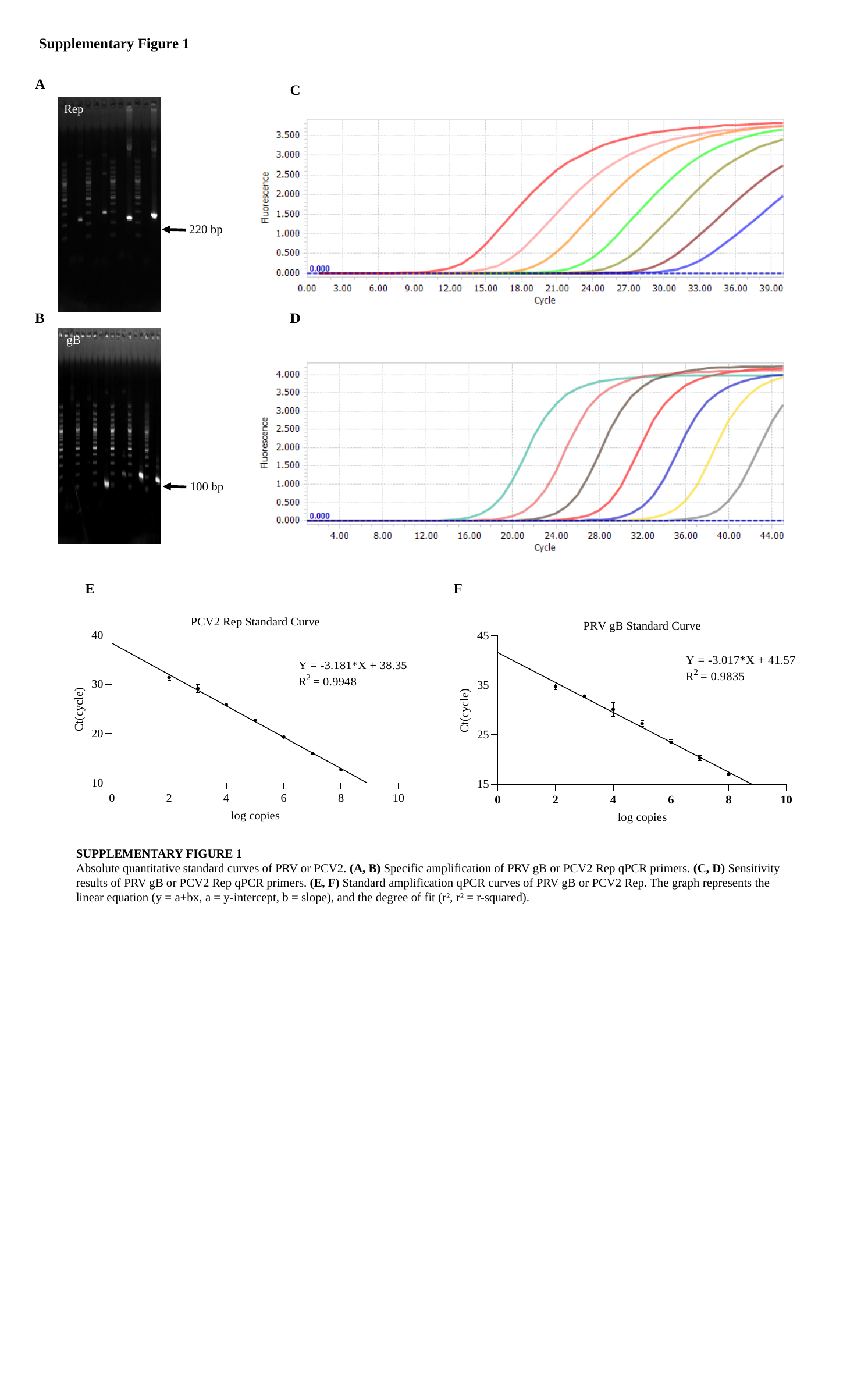

Supplementary Figure 1
A
C
Rep
220 bp
B
D
gB
100 bp
E
F
SUPPLEMENTARY FIGURE 1
Absolute quantitative standard curves of PRV or PCV2. (A, B) Specific amplification of PRV gB or PCV2 Rep qPCR primers. (C, D) Sensitivity results of PRV gB or PCV2 Rep qPCR primers. (E, F) Standard amplification qPCR curves of PRV gB or PCV2 Rep. The graph represents the linear equation (y = a+bx, a = y-intercept, b = slope), and the degree of fit (r², r² = r-squared).

## Slide 2
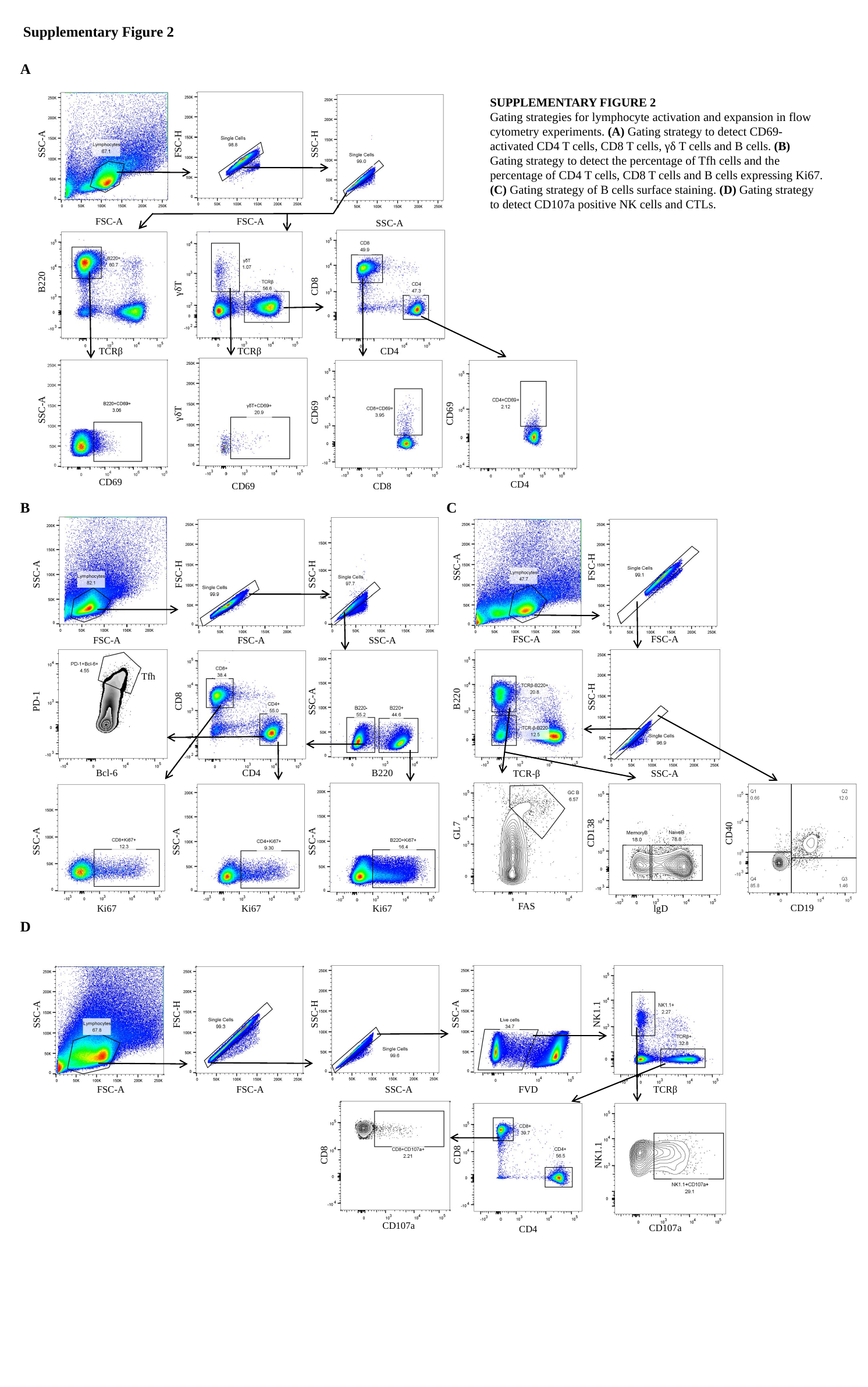

Supplementary Figure 2
A
SSC-A
FSC-H
SSC-H
FSC-A
FSC-A
SSC-A
B220
CD8
γδT
TCRβ
TCRβ
CD4
SSC-A
CD69
γδT
CD69
CD69
CD4
CD69
CD8
SUPPLEMENTARY FIGURE 2
Gating strategies for lymphocyte activation and expansion in flow cytometry experiments. (A) Gating strategy to detect CD69-activated CD4 T cells, CD8 T cells, γδ T cells and B cells. (B) Gating strategy to detect the percentage of Tfh cells and the percentage of CD4 T cells, CD8 T cells and B cells expressing Ki67. (C) Gating strategy of B cells surface staining. (D) Gating strategy to detect CD107a positive NK cells and CTLs.
B
C
SSC-A
FSC-H
SSC-H
FSC-A
FSC-A
SSC-A
Tfh
PD-1
CD8
SSC-A
CD4
Bcl-6
B220
SSC-A
SSC-A
SSC-A
Ki67
Ki67
Ki67
SSC-A
FSC-H
FSC-A
FSC-A
SSC-H
B220
TCR-β
SSC-A
GL7
CD138
CD40
FAS
CD19
lgD
D
SSC-H
SSC-A
NK1.1
SSC-A
FSC-H
FSC-A
FSC-A
SSC-A
FVD
TCRβ
CD8
CD8
NK1.1
CD107a
CD107a
CD4

## Slide 3
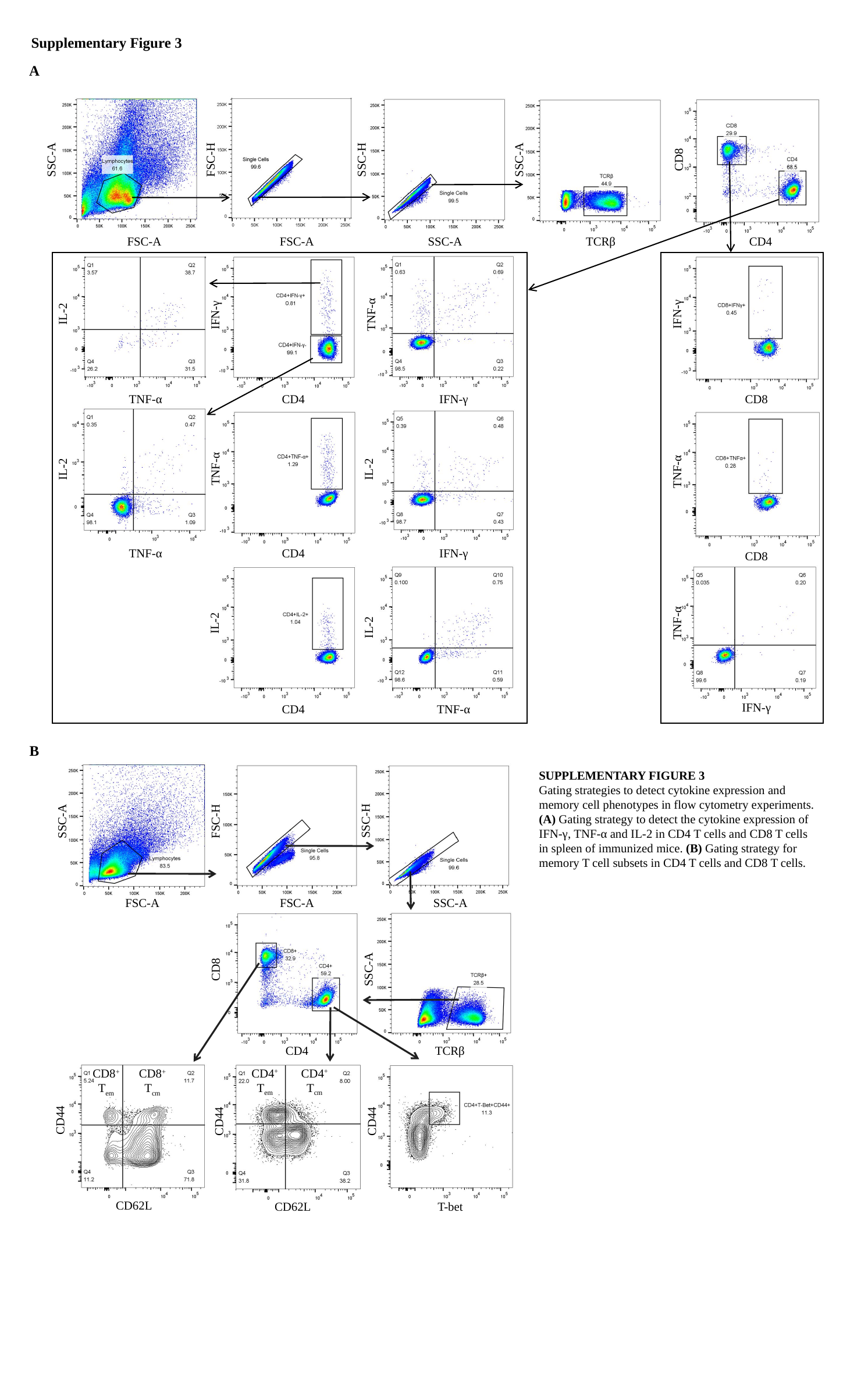

Supplementary Figure 3
A
SSC-A
FSC-H
SSC-H
SSC-A
CD8
FSC-A
FSC-A
SSC-A
TCRβ
CD4
IL-2
IFN-γ
TNF-α
IFN-γ
TNF-α
CD4
IFN-γ
CD8
TNF-α
IL-2
IL-2
TNF-α
TNF-α
CD4
IFN-γ
CD8
IL-2
TNF-α
IL-2
IFN-γ
CD4
TNF-α
B
SUPPLEMENTARY FIGURE 3
Gating strategies to detect cytokine expression and memory cell phenotypes in flow cytometry experiments. (A) Gating strategy to detect the cytokine expression of IFN-γ, TNF-α and IL-2 in CD4 T cells and CD8 T cells in spleen of immunized mice. (B) Gating strategy for memory T cell subsets in CD4 T cells and CD8 T cells.
SSC-H
SSC-A
FSC-H
FSC-A
SSC-A
FSC-A
CD8
SSC-A
CD4
TCRβ
CD4+ Tem
CD4+ Tcm
CD8+ Tem
CD8+ Tcm
CD44
CD44
CD44
CD62L
CD62L
T-bet

## Slide 4
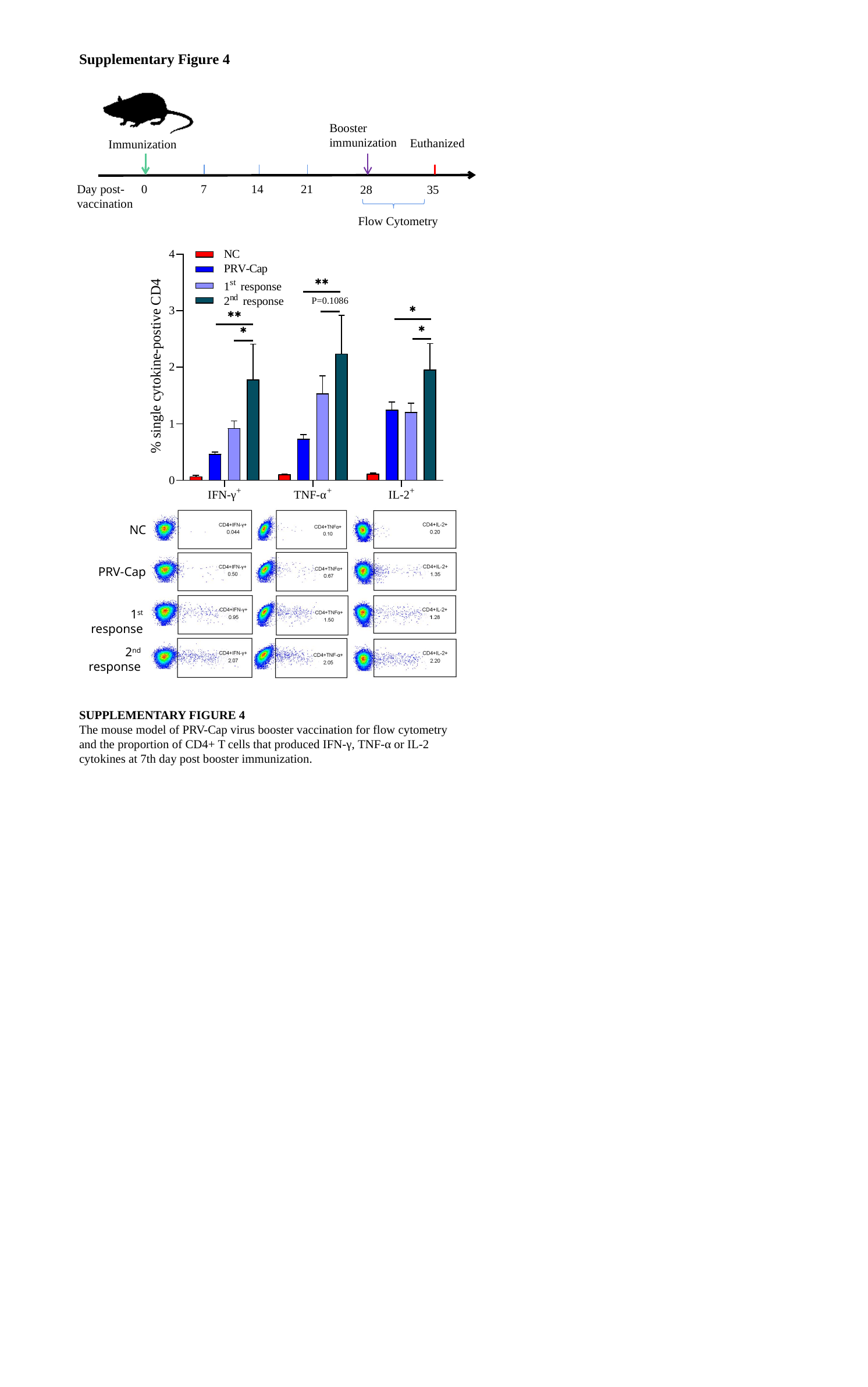

Supplementary Figure 4
Booster immunization
Euthanized
Immunization
0
7
14
21
28
35
Flow Cytometry
Day post-vaccination
NC
PRV-Cap
1st response
2nd response
SUPPLEMENTARY FIGURE 4
The mouse model of PRV-Cap virus booster vaccination for flow cytometry and the proportion of CD4+ T cells that produced IFN-γ, TNF-α or IL-2 cytokines at 7th day post booster immunization.
