# Supplementary material for: Immunological characteristics of a recombinant alphaherpesvirus with an envelope-embedded Cap protein of circovirus
Source: Front Immunol. 2024 Jul 16;15:1438371. doi: 10.3389/fimmu.2024.1438371 (PMC11286414; doi:10.3389/fimmu.2024.1438371)
Supplement: Supplementary file 2 [file Table1.doc]

**Supplementary Material**

| Groups | | Sequence（5’→3’） |
| --- | --- | --- |
| US7-9 | Re-gE-sense | ATCTTCCTGGGCGGGATCGCCT |
|  | Re-gE-antisense | AGATGACCAGCGCGGCGGCGCTGAT |
| PCV2-Rep | Rep-qPCR-F | AGGTGGGTGTTCACTCTG |
|  | Rep-qPCR-R | TGTTCCTTTCGCTTTCTC |
| PRV-gB | gB-qPCR-F0 | ACAAGTTCAAGGCCCACATCTAC |
|  | gB-qPCR-R0 | GTCYGTGAAGCGGTTCGTGAT |

**Supplemental Table 1.** Detection primers of gE and qPCR detection primers of gB and Rep.

**Supplemental Table 2.** Flow cytometry antibody used in this study.

| Name of Abs | Clone | Source& Isotype | Company | Catalog# |
| --- | --- | --- | --- | --- |
| BB700 Rat Anti-Mouse CD4 | RM4-5 | Rat IgG2a.κ | BD | 566407 |
| BV510 Anti-Mouse CD8a | 53-6.7 | Rat IgG2a.κ | Biolegend | 100752 |
| FITC Anti-Mouse TCRγ/ δ | GL3 | Armenian Hamster IgG | Biolegend | 118106 |
| BV421 Anti-Mouse TCRβ chain | H57-597 | Armenian Hamster IgG | Biolegend | 109230 |
| AF700 Anti-Mouse CD45R/B220 | RA3-6B2 | Rat IgG2a.κ | Biolegend | 103224 |
| APC Rat Anti-Mouse IFN-γ | XMG1.2 | Rat IgG1.κ | BD | 554413 |
| FITC Anti-Mouse TNF-α | MP6-XT22 | Rat IgG1.κ | Biolegend | 506304 |
| PE Rat Anti-Mouse IL-2 | JES6-5H4 | Rat IgG2b | BD | 554428 |
| PE-Cy7 Anti-Mouse CD69 | H1.2F3 | Hamster IgG1, λ3 | BD | 552879 |
| FITC Anti-Mouse/human CD44 | IM7 | Rat IgG2b.κ | Biolegend | 103006 |
| PE/Cy7 Anti-Mouse CD62L | MEL-14 | Rat IgG2a.κ | Biolegend | 104418 |
| PE Mouse anti-T-bet | O4-46 | Mouse IgG1.κ | BD | 561268 |
| BV421 Mouse Anti-Ki67 | B56 | Mouse IgG1.κ | BD | 565929 |
| PE Mouse anti-Bcl-6 | K112-91 | Mouse IgG1.κ | BD | 561522 |
| APC anti-mouse CD279(PD-1) | 29F.1A12 | Rat IgG2a.κ | Biolegend | 135209 |
| PE/Cy7 Anti-Mouse CD107a | 1D4B | Rat IgG2a.κ | Biolegend | 121620 |
| PE/Cy7 Rat Anti-Mouse CD19 | 1D3 | Rat IgG2a.κ | BD | 557655 |
| APC Rat anti-Mouse CD40 | 3/23 | Rat IgG2a.κ | BD | 558695 |
| PerCP-Cy5.5 Rat Anti-Mouse IgD | 11-26c.2a | Rat IgG2a.κ | BD | 564273 |
| Rat Anti-Mouse CD138 | 281-2 | Rat IgG2a.κ | BD | 563192 |
| PE anti-mouse/human GL7 | GL7 | Rat IgM.κ | Biolegend | 144608 |
| BV711 Hamster Anti-Mouse CD95 | Jo2 | Hamster IgG2.λ2 | BD | 740716 |
| FITC Rat Anti-Mouse CD45R | RA3-6B2 | Rat IgG2a.κ | BD | 553087 |
| Fixable Viability Dye eFluor™ 780 |  |  | eBioscience | 65-0865-14 |

**Supplemental Figure Legends**

**SUPPLEMENTARY FIGURE 1**

Absolute quantitative standard curves of PRV or PCV2. **(A, B)** Specific amplification of PRV gB or PCV2 Rep qPCR primers. **(C, D)** Sensitivity results of PRV gB or PCV2 Rep qPCR primers. **(E, F)** Standard amplification qPCR curves of PRV gB or PCV2 Rep. The graph represents the linear equation (y = a+bx, a = y-intercept, b = slope), and the degree of fit (r², r² = r-squared).

**SUPPLEMENTARY FIGURE 2**

Gating strategies for lymphocyte activation and expansion in flow cytometry experiments. **(A)** Gating strategy to detect CD69-activated CD4 T cells, CD8 T cells, γδ T cells and B cells. **(B)** Gating strategy to detect the percentage of Tfh cells and the percentage of CD4 T cells, CD8 T cells and B cells expressing Ki67. **(C)** Gating strategy of B cells surface staining. **(D)** Gating strategy to detect CD107a positive NK cells and CTLs.

**SUPPLEMENTARY FIGURE 3**

Gating strategies to detect cytokine expression and memory cell phenotypes in flow cytometry experiments. **(A)** Gating strategy to detect the cytokine expression of IFN-γ, TNF-α and IL-2 in CD4 T cells and CD8 T cells in spleen of immunized mice. **(B)** Gating strategy for memory T cell subsets in CD4 T cells and CD8 T cells.

**SUPPLEMENTARY FIGURE 4**

The mouse model of PRV-Cap virus booster vaccination for flow cytometry and the proportion of CD4^+^ T cells that produced IFN-γ, TNF-α or IL-2 cytokines at 7^th^ day post booster immunization.
